# Impact of Music Therapy on Quality of Life in Children with Cancer

**DOI:** 10.3390/children10091486

**Published:** 2023-08-31

**Authors:** Faten Fedhila, Mohamed Wassim Hannachi, Elhem Jbebli, Ines Selmi, Samar Rhayem, Issam Magouri, Hedia Bellali, Monia Khemiri

**Affiliations:** 1 Pediatric “A” Department of the Bechir Hamza Children’s Hospital, 169 Boulevard 9 April 1938, Tunis 1006, Tunisia; med.wassim.hannachi@gmail.com (M.W.H.); elhem.jbebli@fmt.utn.tn (E.J.); samar.rhaiem@fmt.utn.tn (S.R.); issam.magouri@gmail.com (I.M.); monia.khemiri@fmt.utn.tn (M.K.); 2 Pediatrics Department, Mongi Slim Hospital, Sidi Daoud La Marsa, Tunis 2046, Tunisia; ines.selmi@fmt.utn.tn; 3 Clinical Epidemiology Department, Hbib Thameur Hospital, 3 Street A. Ben Ayed 1089, Tunis Chebbi, Tunis 1008, Tunisia; hedia.bellali@fmt.utn.tn

**Keywords:** children, cancer, oncology, music therapy, quality of life

## Abstract

Background: Music therapy (MT) is a non-pharmacological treatment increasingly used to reduce stress and anxiety in hospitalized children affected by cancers. The aim of this study was to evaluate the impact of MT on quality of life in children with cancer and determine its effect on cardiorespiratory rates. Methods: We conducted a quasi-experimental study between 1 April and 31 August 2021 at Bechir Hamza children’s Hospital in Tunis, including children treated for cancer. The child or parent completed the PedsQL Module Cancer French version 3.0 questionnaires before and after four weekly music therapy sessions. The child’s respiratory and heart rates were measured before and after each session. Results: We included 20 children whose mean age was 7 ± 4.5 years. The median value of the total questionnaire score increased from 57 [46; 70] to 72 [67; 85] (*p* < 10^−3^) noting a significant reduction in pain (*p* = 0.02), nausea (*p* = 0.009), and anxiety related to medical procedures (*p* = 0.009) and worry about the future (*p* = 0.005). We highlighted a significant decrease in respiratory and heart rate after MT (*p* < 0.05). Conclusions: MT has positive impact on quality of life in children with cancer and reduces stress by lowering their cardiorespiratory rates.

## 1. Introduction

The care of a child with cancer involves several physical and psychological constraints that can be perceived as “aggression” by the child and their family. This issue can cause negative reactions, such as non-adherence to treatment or even a depressed mood and aggressive behavior toward the medical staff [1]. Also, two-thirds of childhood cancer survivors experience long-term adverse health effects from their illness or its treatment [2]. Hence, long-term goals in the care and treatment for a child with cancer include not only curing the patient, but also ensuring that they have a satisfactory health-related quality of life (QoL).

Health-related QoL is defined as a multidimensional assessment of physical, psychological, and social functioning and sensitive to developmental changes in children and adolescents [3]. Health-related QoL is increasingly being used as an outcome metric in clinical trials, as well as to monitor the occurrence of sequelae in childhood cancer survivors [4].

Among the non-pharmacological measures used to help children with cancer and their families to better cope with this situation of extreme stress, music therapy (MT) has shown an ability to improve the quality of life of children with cancer [5].

According to the French Federation of Music Therapy, MT is “a practice of care, counseling, accompaniment, support or rehabilitation, using sound and music, in all their forms, as a means of expression, communication, structuring and analysis of the relationship” [6]. It is said to be “active” when it favors sound and musical production, improvisation and creativity, and “receptive” when it is based more on listening. Since the first use of MT in the 1970s, this process has occupied an increasingly important position, all over the world, in the psychological and therapeutic care of patients with serious pathologies.

To our knowledge, there is no prior Tunisian publication regarding MT and its effects on children with cancer. The aim of our study was mainly to evaluate the impact of MT on quality of life in children hospitalized with pediatric cancers and, secondarily, to investigate its effect on cardiac and respiratory parameters.

## 2. Materials and Methods

### 2.1. Study Design

We conducted a quasi-experimental study (before–after type) comparing the patient before and after MT sessions from 1 April to 31 August 2021 at the pediatric oncology unit of Bechir Hamza Children’s Hospital in Tunis.

### 2.2. Inclusion and Exclusion Criteria

Patients aged 2 to 14 years old who were hospitalized on 1 April 2021 were included. 

Children with deafness, profound encephalopathy, or impaired consciousness were not included. 

Children who died or who presented complications altering their state of consciousness or their hearing during the study period were excluded.

### 2.3. Procedures

Firstly, PedsQL Module Cancer questionnaire French version 3.0 [7] was translated into the Tunisian dialect and filled out by the child or their parent. The questionnaire was composed of 27 items divided into 8 dimensions: pain, nausea, anxiety related to medical procedures, anxiety related to treatments, worry, cognitive disorders, perception of physical appearance, and communication. For each item, the respondent answered questions, and the values assigned to the different items were transformed into a score. The overall score corresponded to the average value of the eight scores obtained. The higher the score, the better the quality of life. For example, if the anxiety score of the questionnaire increased, the patient was less anxious.

Secondly, the patient individually participated in four MT sessions once per week for about 20 min. The type of MT (active or passive) was decided according to the child’s preferences in agreement with the music therapist. The therapist adapted the music played to the age and tastes of the child, and it had varied sounds (traditional, classical, modern, or a combination thereof). The sessions were conducted by the same music therapist in a newly designed MT workshop at the department. Each child’s respiratory rate (RR) and heart rate (HR) were recorded using study sheets by a physician, who was not involved in the study, before and at the end of each MT session. The patient was at rest for 15 min before we measured their cardiorespiratory parameters. 

Thirdly, the same questionnaire was filled out by the patient or their parent after the four MT sessions. Data collection was performed using two informative sheets. The first sheet, which was completed by a doctor who was not part of the medical team, mainly focused on the sociodemographic and clinical data of the patient. The second sheet was filled out by the music therapist, who specified the type of MT, the scale played, and the instruments used.

### 2.4. Statistical Methods

SPSS Statistics version 23.0 software was used for data entry and analysis. 

Qualitative variables were expressed in terms of proportions. 

Quantitative variables were described in terms of means, medians, and standard deviations. 

We used non-parametric tests, such as the Wilcoxon ranking test to compare medians, the Mann–Whitney test to compare means, and the Kolmogorov–Smirnov test to verify the normality of the distribution. 

The significance threshold (*p* value) was set at 0.05.

### 2.5. Ethical Issues

After obtaining adequate and complete information, free and informed consent was obtained from the children’s parents. Prior approval from the Ethics Committee of Bechir Hamza Children’s Hospital was given before the start of the trial. 

## 3. Results

### 3.1. Sociodemographic Data 

**Figure 1:**
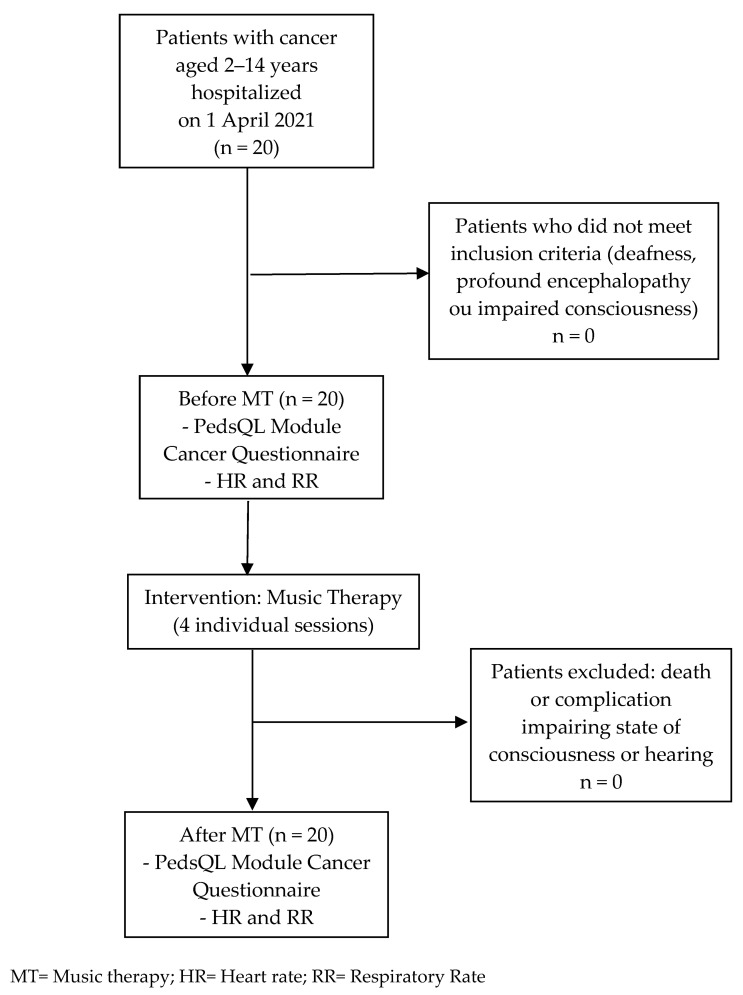
Recruitment flowchart of the study.

All of the patients aged 2–14 years old who were hospitalized on 1 April 2021 met the inclusion criteria. No patient was excluded during the study (Figure 1).

The sex ratio was 1/1. The average age was 7 ± 4.5 years, with a minimum age of 2 years old and a maximum age of 14 years old. 

Among the children included in the study, 15 had at least one sibling, 8 were in school during the study period, and 2 who were frequently hospitalized or received daily care unfortunately had their schooling interrupted. The other children were not in the age range required to attend school (*n* = 10) (age < 6 years).

### 3.2. Disease Data

The cancers collected in our study were hematological malignancies in three cases and solid tumors in 17 cases. Cancer was metastatic in 14 patients.

The majority of children were undergoing treatment (16/20), though two patients were in relapse, one patient was newly diagnosed, and one was receiving palliative treatment.

Nine patients had been cared for in the unit for more than six months (9/20).

The majority of children were admitted for chemotherapy treatment (12/20), though six patients were admitted for febrile aplasia (6/20) and two patients were admitted for acute fever (2/20).

### 3.3. Music Therapy Data

Sessions 1–3 were mostly receptive, while Session 4 was both receptive and active (Table 1). The scales played were more or less balanced between the participants during Sessions 1, 2, and 4, while in Session 3, the most played scales were minor and mixed. Different instruments were used (shaker, kalimba, piano, guitar, maraca, ukulele, etc.), though the most frequently used instrument was the guitar (Table 1).

### 3.4. Assessment of Each Patient’s Quality of Life

**Figure 2:**
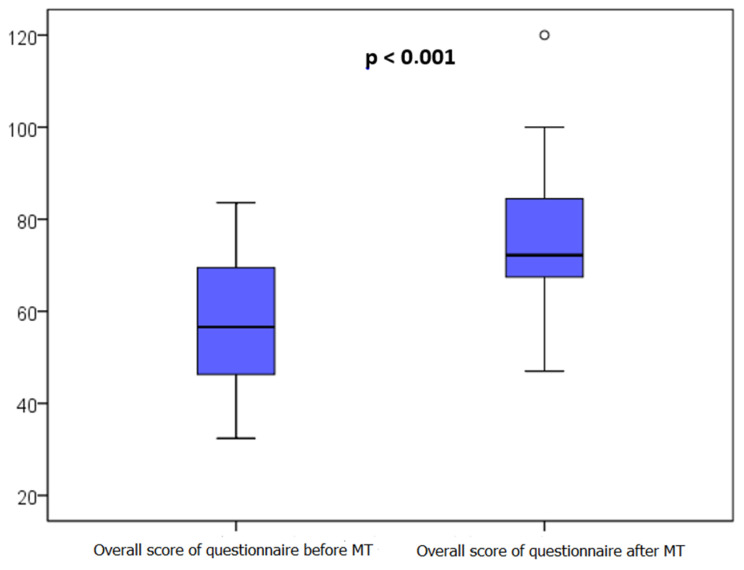
Evolution in the overall score of the PedsQL questionnaire before and after four music therapy sessions.

The PedsQL Module Cancer questionnaire 3.0 was filled out in 13 cases by one of the parents and in 7 cases by the child themself. The median value of the total score increased by 21.6% (*p* < 10^−3^) from 57 (46–70) before MT to 72 (67–85) after four sessions (Figure 2). We also noted a significant decrease in pain (*p* = 0.02), nausea (*p* = 0.009), anxiety related to medical procedures (*p* = 0.009), and anxiety related to treatment (*p* = 0.03), as well as concerns about the future (*p* = 0.005). 

Furthermore, we reported a significant improvement in each child’s physical perception (*p* = 0.01), as well as in the communication between the patient and their family members and medical staff (*p* = 0.005) (Table 2). However, there was no significant improvement in cognitive disorders (*p* = not significant (NS)).

### 3.5. Analytical Study

We noted a significant improvement in the total scores for both sexes (Table 3). Boys had less nausea (*p* = 0.03), less anxiety related to medical procedures (*p* = 0.008), less worry (*p* = 0.02), and a better physical perception (*p* = 0.03) after four sessions of MT. For girls, overall quality of life also significantly improved after the MT sessions (*p* = 0.03), though their responses were less significant than those of the boys (Table 3). On the other hand, communication has improved (*p* = 0.03) for girls, unlike for the boys (*p* = NS). Using the Mann–Whitney U test of independent samples, we found a significant difference in the worry score between boys and girls: boys were less worried than girls after the MT sessions (*p* = 0.02).

Looking at different age groups, the two groups that responded best to MT were those aged between 2 and 4 years old (*p* = 0.03) and those aged between 8 and 14 years old (*p* = 0.02) (Table 3). Anxiety decreased for these two groups, but did so more significantly for the group aged 8-to-14 years old (*p* = 0.02). Worry only significantly decreased in the 2–4-year-old group (*p* = 0.04). Using the Kruskal–Wallis test of independent samples, we did not find statistically significant differences between age groups regarding the different scores on the questionnaire after MT.

Taking into account the family situation, the group of children (n = 5) without siblings were not sensitive to music therapy, unlike those with siblings (Table 3). Nausea (*p* = 0.01), anxiety related to medical procedures (*p* = 0.02), anxiety related to and treatment (*p* = 0.03), and worry (*p* = 0.008) decreased, whereas communication (*p* = 0.009) improved in the sibling group. Using the Mann–Whitney U test of independent samples, we found a statistically significant difference between children with siblings and children without siblings regarding the total questionnaire score (*p* = 0.005), the anxiety related to medical procedures (*p* = 0.008), the anxiety score related to treatment (*p* = 0.03), and the communication with the medical staff (*p* < 10^−3^).

On the other hand, our study demonstrated that patients with metastatic tumors had a better response to MT (Table 3), with a significant increase in overall quality of life scores (*p* = 0.003), as well as improved communication (*p* = 0.003) and a significant decrease in nausea (*p* = 0.002), treatment-related anxiety (*p* = 0.03), and worry (*p* = 0.03). For children with a localized tumor, the improvement was notable for anxiety scores related to medical procedures (*p* = 0.04). Nevertheless, we did not find a statistically significant difference between the two groups according to the Mann–Whitney U test of independent samples. 

Whatever the duration of care within the service, the quality of life of the participants improved after four MT sessions, though the children treated recently (less than six months) seemed to respond better to MT (Table 3). According to the Mann–Whitney U test of independent samples, children who had received care for less than six months developed better self-esteem than other children (*p* = 0.02).

By focusing on schooling, our study showed that schooled children were not sensitive to MT, unlike non-schooled children (Table 3). These results should be interpreted with caution because age can be a confounding factor, since the majority of non-schooled children are too young to be schooled (age < 6 years). Nevertheless, we did not find a significant difference between children according to their school status or age (age ≥ 6 years or <6 years).

### 3.6. Evaluation of Cardiorespiratory Parameters

We observed a significant decrease in RR and HR in all participants after each MT session (Table 4). According to the Mann–Whitney U test of independent samples, these parameters were not affected by gender, age, education, siblings, stage of disease, or the duration of care.

## 4. Discussion

Our study demonstrated a significant improvement in quality of life in children with cancer after MT, which has already been reported by several authors. Thus, a recent meta-analysis [5] of eleven articles including 429 children aged between 0 and 18 years old showed that music positively influenced the quality of life of children compared to the control groups.

### 4.1. Music Therapy and Pain

Our results also suggested that pain experienced by children significantly decreased after MT. A randomized clinical trial [8] was carried out in 40 children aged 7-to-12 years old with leukemia and compared the intensity of pain induced via a lumbar puncture between two groups: a control group (*n* = 20) and a group that received MT (*n* = 20). Anxiety scores were measured before and after the procedure. The results showed lower pain scores and lower heart and respiratory rates in the music group during and after the lumbar puncture. Anxiety scores were also lower in the music group before and after the procedure. The authors concluded that MT is a reliable and inexpensive means of distraction that reduced the level of anxiety in children with cancer before invasive procedures and reduces the intensity of pain at the time of the procedure. 

These results were supported by those of Da Silva Santa’s meta-analysis [5], which demonstrated that MT had a positive impact on anxiety and pain in the group of patients who had MT sessions compared to the control group. Dobeck et al. [9] attempted to analyze the effects of MT on the central nervous system using magnetic resonance imaging. They proved that emotions provoked by music affected the neuronal activity of the brainstem and the spinal cord, while the intensity of pain significantly decreased when MT was concomitantly performed in tandem with the painful stimuli.

### 4.2. Music Therapy and Anxiety

It was demonstrated in our study that the level of anxiety significantly decreased after four sessions of MT, which has been reported in various studies, particularly in adults. In fact, a meta-analysis including 81 trials with 5567 participants, which included 5306 adults and 270 children, has shown that MT reduced anxiety and depression in adults with cancer [10].

### 4.3. Music Therapy, Self-Esteem, and Communication

Our results also showed the effects of MT on self-esteem, which have already been reported by a multicenter study conducted in Northern Ireland by Porter et al. [11], which included 251 children aged 8-to-16 years old with social, behavioral and emotional difficulties. They were divided into two groups, with one group receiving 12 weekly MT sessions and another group receiving usual care. The evaluation at the 13th week showed a significant decrease in depressive symptoms and a marked improvement in self-esteem in the group with MT, as well as an improvement in communication for children over 13 years old (*p* = 0.007). In fact, the child affected by cancer saw his body metamorphose more or less rapidly, hence generating a potential risk of damage to self-esteem, particularly among adolescents. MT could, therefore, be a good alternative strategy to improve self-perception and communication with people among children. 

An American multicenter randomized control trial [12] studied the impact of MT on the social behavior of 83 children with cancer aged 4–7 years old by comparing a group treated with MT (*n* = 27) to a group that listened to music (*n* = 28) and another group that listened to audio storybooks (*n* = 28). MT improved facial expressions and enhanced active engagement in social relationships (*p* < 0.0001), as well as social interactivity (*p* < 0.05). These positive repercussions would facilitate communication with family and medical staff, especially among adolescents with cancer, who tend to isolate themselves from the outside world.

### 4.4. Music Therapy and Cognitive Impairment

Our study did not demonstrate the efficacy of MT in improving cognitive impairment. This issue could be explained by the low prevalence of cognitive disorders in our study population (cognitive impairment scores before MT are shown in Table 2). Also, studies of the effects of MT on these pathologies have mainly been carried out on specific populations: adults or children suffering from severe neuropsychological disorders, such as autism [13]. 

A meta-analysis [14] of the impact of MT on the treatment of schizophrenia, which was published in 2017 and includes 18 studies with a total of 1215 participants, highlighted the short-, medium-, and long-term beneficial effects of MT on the behavior of schizophrenic patients. The data analyzed found positive effects of MT on the quality of life and social functioning of patients who received MT compared to the control group [14]. Regularly listening to music reduced auditory hallucinations in these patients and improved their quality of life. The positive effect of music on the brain in schizophrenia has also been demonstrated via functional magnetic resonance imaging [14]. 

Similarly, a Chinese study published in 2018 revealed a decrease in the incidence of psychiatric disorders in adults with dementia and Alzheimer’s disease [15].

### 4.5. Music Therapy and Cardiorespiratory Rates

The effects of MT on the cardiorespiratory parameters demonstrated in our study have also been reported by other authors. 

Uggla et al. [16] conducted a randomized control trial involving 24 children who had received a hematopoietic stem cell transplant. They observed a significant drop in lasting heart rate for four-to-eight hours after the intervention, with no effect on the respiratory rate and oxygen saturation. They also reported the positive experiences of children who were confronted with MT, identifying a particular impact on well-being and self-confidence [17]. In another study of 115 adult patients [18] for whom music was played in the operating room, we compared the vital parameters measured in the waiting room without music to those measured in the operating room with music. Mean arterial pressure, HR, and RR were significantly lower in the operating room with music (*p* < 0.0001).

A systematic review of the literature [19] showed that listening to music stimulated the parasympathetic tone of the cardiovascular system.

### 4.6. Strengths and Limitations

Among the strengths of our study, we cite its innovative nature, highlighting a technique that is still little known in Tunisia, but which has already proven its worth in developed countries in improving the daily lives of children with cancer. This study is the first of its kind in North Africa. It is a quasi-experimental study in which the patient has control, thus avoiding the bias of interindividual variability. We also highlight the presence of a trained music therapist. Nikjeh et al. [20] found that musicians trained in MT performed better than those who were not trained in MT. 

Among the weak points, we report the small number of participants and the absence of a control group, which could have increased the power of the study and the relevance of the results. By comparing the same patient before and after the intervention, we may have introduced measurement and subjectivity bias because the child may have been enthusiastic about the idea of participating in the different MT sessions, thus making their judgment more subjective. The PedsQL questionnaire was translated into the Tunisian dialect by a Tunisian doctor, which should be followed by a back-translation procedure in order to create the best cross-cultural adaptation of the questionnaire and a validated translation equivalent to the original version.

### 4.7. Future Research Implications

Further research into MT and its effects on paediatric and adolescent oncology patients is required. We believe that future studies must have a greater sample size and appropriate methodological quality.

### 4.8. Practical Clinical Implications

MT is an easy and non-invasive tool that should be part of the daily care of children suffering from severe diseases. However, it can be expensive and time consuming, and it involves the presence of a trained and motivated music therapist.

## 5. Conclusions

Our study showed a positive impact of MT on quality of life in children affected by cancer. It also reduced anxiety and stress symptoms by lowering the heart and respiratory rates. These results suggest the importance of integrating MT into daily practice in pediatric oncology. However, randomized double-blind studies with larger samples are needed to confirm these results.

## Figures and Tables

**Table 1:** Characteristics of the different music therapy sessions.

		Session 1	Session 2	Session 3	Session 4
Type of session (number)	Receptive	15	11	12	6
	Active	1	2	2	4
	Receptive and active	4	7	6	10
Musical scale played (number)	Major	9	8	2	6
	Minor	6	5	9	7
	Major and minor (mixed)	5	7	9	7
Average duration (min)		22 ± 8 (15–45)	21 ± 6 (14–40)	20 ± 6 (15–40)	24 ± 7 (15–40)
**Most commonly used musical instrument (number)**		Guitar 9	Guitar 11	Guitar 15	Guitar 9

Min: minutes.

**Table 2:** Cancer Module PedsQL scores before and after music therapy.

	Before MT Median [IQR]	After MT * Median [IQR]	*p* **
Total Cancer Score Module	57 [46; 70]	72 [67; 85]	<10^−3^
Pain score	81 [28; 100]	100 [56; 100]	0.02
Nausea score	53 [38; 70]	73 [46; 89]	0.009
**Anxiety score related to medical procedures**	33 [10; 56]	58 [42; 86]	0.009
Treatment-related anxiety score	67 [17; 81]	67 [50; 92]	0.03
Worry score	50 [33; 67]	75 [58; 90]	0.005
Cognitive impairment score	80 [61; 100]	84 [75; 93]	NS
Physical appearance perception score	67 [27; 100]	83 [67; 100]	0.01
Communication score	63 [17; 81]	83 [77; 100]	0.005

* after four music therapy sessions; ** *p*-value according to the Wilcoxon test of two related samples. MT = music therapy; IQR = interquartile range; NS = not significant.

**Table 3:** Scores of the PedsQL cancer module questionnaire before and after music therapy according to different parameters.

Overall Score	Before MT Median [IQR]	After MT * Median [IQR]	*p* **
Gender:			
Boys	59 [46; 69]	75 [71; 84]	0.005
Girls	56 [44; 77]	68 [55; 96]	0.03
Age:			
2–4 years	61 [39; 75]	76 [62; 89]	0.03
5–7 years	55 [44;75]	69 [59; 83]	0.14
8–14 years	57 [46; 70]	74 [69; 83]	0.02
Siblings:			
Have siblings	61 [51; 75]	76 [71; 89]	0.001
No siblings	45 [36; 60]	62 [49; 68]	0.2
School:			
Schooled children	61 [47; 70]	71 [59; 81]	0.06
Non-schooled children	56 [41; 73]	74 [68; 88]	0.003
Disease stage:			
Localised	61 [52; 69]	77 [65;86]	0.08
Metastatic	56 [40; 72]	70 [66; 86]	0.003
Duration of care			
<6 months	65 [54; 75]	76 [68; 89]	0.006
≥6 months	51 [40; 61]	69 [59; 77]	0.02

* after four music therapy sessions; ** *p*-value according to the Wilcoxon test of two related samples. MT = music therapy; IQR = interquartile rang.

**Table 4:** Respiratory and heart rates of children before and after music therapy.

	Session 1			Session 2			Session 3			Session 4		
	Before MT	After MT	*p*	Before MT	After MT	*p*	Before MT	After MT	*p*	Before MT	After MT	*p*
RR median IQR	25 [22; 32]	20 [20; 27]	0.003	24 [22; 33]	24 [20; 32]	0.05	27 [22; 34]	23 [20; 28]	0.004	27 [24; 32]	22 [20; 28]	<10^−3^
HR median IQR	120 [102; 133]	105 [89; 122]	0.01	119 [111; 130]	113 [92; 124]	0.005	115 [102; 131]	103 [87; 127]	<10^−3^	105 [97; 120]	95 [87; 101]	<10^−3^

RR: Respiratory rate; HR: Heart Rate; MT = music therapy; IQR = interquartile range; *p* = *p*-value according to the Wilcoxon test of two related samples.

## Data Availability

The data presented in this study are available on request from the corresponding author. The data are not publicly available due to ethical restrictions.
